# Evaluating SARS-CoV-2 Saliva and Dried Blood Spot Surveillance Strategies in a Congregate Population 

**DOI:** 10.3201/eid2909.230417

**Published:** 2023-09

**Authors:** Liana R. Andronescu, Stephanie A. Richard, Eric D. Laing, Nora Pisanic, Si’Ana A. Coggins, Magdielis Gregory Rivera, Kate Kruczynski, Adam K. Saperstein, Jitendrakumar Modi, Jamie A. Fraser, Saira Shaikh, Christopher C. Broder, Timothy H. Burgess, Christopher D. Heaney, Simon D. Pollett, Eugene Millar, Christian L. Coles, Mark P. Simons

**Affiliations:** Uniformed Services University of the Health Sciences, Bethesda, Maryland, USA (L.R. Andronescu, S.A. Richard, E.D. Laing, S.A. Coggins, A.K. Saperstein, J.A. Fraser, S. Shaikh, C.C. Broder, T.H. Burgess, S.D. Pollett, E. Millar, C.L. Coles, M.P. Simons);; The Henry M. Jackson Foundation for the Advancement of Military Medicine, Inc., Bethesda (L.R. Andronescu, S.A. Richard, S.A. Coggins, J.A. Fraser, S. Shaikh, S.D. Pollett, E. Millar, C.L. Coles);; Johns Hopkins University, Baltimore, Maryland, USA (N. Pisanic, M. Gregory Rivera, K. Kruczynski, C.D. Heaney);; Naval Health Clinic, Annapolis, Maryland, USA (A.K. Saperstein, J. Modi)

**Keywords:** COVID-19, coronavirus disease, SARS-CoV-2, severe acute respiratory syndrome coronavirus 2, viruses, respiratory infections, zoonoses, vaccine-preventable diseases, saliva, dried blood spot, surveillance, strategies, congregate population

## Abstract

The optimal approach to COVID-19 surveillance in congregate populations remains unclear. Our study at the US Naval Academy in Annapolis, Maryland, USA, assessed the concordance of antibody prevalence in longitudinally collected dried blood spots and saliva in a setting of frequent PCR-based testing. Our findings highlight the utility of salivary-based surveillance.

Congregate populations, including those in university and military settings, are at high risk for SARS-CoV-2 transmission because of crowding, frequent physical contact, and environmental contamination (*1*). Using self-collected saliva for surveillance may be a noninvasive alternative to serum and warrants further evaluation to guide population surveillance strategies.

Assessment of SARS-CoV-2 infection prevalence often is underestimated because of asymptomatic and paucisymptomatic infections that are not often captured by screening test strategies (*2*–*4*), but those infections contribute to high attack rates in congregate populations (*5*–*9*). This study evaluated the use of saliva to estimate the prevalence of SARS-CoV-2 infection among a congregate population of young and initially immunologically naive adults at the US Naval Academy (USNA) in Annapolis, Maryland, USA.

## The Study

The Observational Seroepidemiologic Study of COVID-19 at the USNA (TOSCANA) study enrolled male and female midshipmen to estimate the SARS-CoV-2 attack rate and assess the concordance of seroprevalence between blood and saliva. All midshipmen at USNA (≈4,500) reside in a single dormitory. During the time of the study, nonpharmaceutical interventions included mask wearing, weekly PCR-based surveillance, and isolation of cases (Appendix). Pharmaceutical interventions included receipt of the Moderna (https://www.modernatx.com) SARS-CoV-2 mRNA vaccine in March 2021 (first dose) and April 2021 (second dose); >96% of all midshipmen had documented receipt of 2 doses.

We initiated the process of recruiting, enrolling, and acquiring consent of participants at the start of the academic year. After providing consent, participants completed the baseline questionnaire regarding demographic information, risk factors for acute respiratory infection, and previous infections or exposures to SARS-CoV-2. Paired self-collected saliva and dried blood spots were collected at enrollment (August 2020, visit 1 [V1]) and follow-up visits in December 2020 (V2), February 2021 (V3, saliva only), and April–May 2021 (V4) (Appendix Table).

Methods for dried blood spot (DBS) collection and testing has been described previously (*10*). We collected blood samples by using the Mitra Blood Collection Kit (Neoteryx, https://www.neoteryx.com) and tested them for SARS-CoV-2 reactive IgG by using an in-house multiplex microsphere-based immunoassay. The antigenic targets were a prefusion-stabilized SARS-CoV-2 spike glycoprotein ectodomain trimer and a nucleocapsid protein; we detected antigen-specific IgG levels by using a Bio-Plex 200 HTF multiplexing systems (Bio-Rad, https://www.bio-rad.com) and reported results as median fluorescence intensity (MFI).

We collected saliva samples by using an Oracol S14 collection device (Malvern Medical Developments, https://www.malmed.co.uk) and tested them as previously described (*11*). We tested samples for IgG binding to any of 7 SARS-CoV-2 antigen components (2 SARS-CoV-2 nucleocapsid proteins, 3 receptor-binding domain [RBD] proteins, and 2 spike proteins) by using a multiplex immunoassay. After background subtraction, we classified samples positive for RBD and nucleocapsid IgG as indicative of prior infection, whereas we classified samples positive for only RBD IgG as indicative of SARS-CoV-2 vaccination.

As part of routine clinical care, USNA’s Brigade Medical Clinic collected nasopharyngeal swab specimens from all returning midshipmen in August and throughout the school year when they visited the clinic with symptoms of respiratory illness. In addition, each week we randomly selected 15% of the asymptomatic midshipmen population for reverse transcription PCR (RT-PCR) screening; we also tested 100% of in-season varsity athletes each week. We excluded from weekly testing all participants who had confirmed positive SARS-CoV-2 infection during the preceding 90 days. We tested nasopharyngeal swab samples by using SARS-CoV-2 RT-PCR and made results accessible through electronic medical records.

We compared seroconversion rates with cumulative frequencies of molecularly confirmed infections. We calculated correlation coefficients for spike IgG and nucleocapsid IgG MFI in saliva and DBS. We used the Cohen kappa coefficient (κ) to measure concordance of saliva with DBS nucleocapsid IgG and spike IgG positivity and to measure concordance of PCR tests with seroconversions.

This study was approved by the Uniformed Services University Institutional Review Board under protocol IDCRP-129. All participants provided written informed consent.

In August 2020, a total of 104 midshipmen enrolled in the study; 64.4% were men, 92.3% were white, 8 (7.7%) reported COVID-19 exposure, and 11 (10.6%) reporting a COVID-19 diagnosis before arrival at USNA. At baseline, 17 (16%) participants showed evidence of SARS-CoV-2 infection based on spike IgG values in DBS.

Among the participants who were serologically negative for SARS-CoV-2 at enrollment, 18 seroconversions were detected in saliva, 19 were detected in DBS, and 19 were detected by PCR by the end of follow-up (Table 1); however, at V4 (postvaccination), additional cases were detected by DBS and saliva that were missed by PCR testing. One participant had a positive PCR result before a serologic result; the PCR test was conducted in August 2020, and the participant had no record of seroconversion through the end of the study.

**Table 1:** New SARS-CoV-2 infections detected among 79 study participants, by PCR and serologic test, at each specimen collection timepoint, US Naval Academy, Annapolis, Maryland, USA, August 2020–May 2021*

Test	2020 Aug (V1)	2020 Dec (V2)	2021 Feb (V3)	2021 May (V4)†	Total
Saliva seroconversion‡	0	2	3	13	18
Dried blood spot seroconversion	0	3	NA	16	19
PCR-positive	1	3	5	10	19

*Sample restricted to participants who had a PCR test on record (from screening or medically attended SARS-CoV-2) and were not seropositive at the first visit in August 2020. V1, V2, V3, and V4 note the visit timepoint that matches to the corresponding month. †Collection time is postvaccination; nucleocapsid IgG and not spike IgG seroconversion alone was used to measure infection. ‡Salivary nucleocapsid IgG positivity defined as receptor-binding domain IgG and nucleocapsid IgG positive; salivary spike IgG positivity used a receptor-binding domain target.

By V4, 100% of remaining participants were spike IgG seropositive, and 49.1% of remaining participants with both DBS and saliva seroconverted to nucleocapsid IgG as evaluated by DBS (Table 2). Among participants with both DBS and saliva samples (n = 55), spike IgG results had an observed agreement of 0.85 and a κof 0.49 (95% CI 0.15–0.83) at V1. By V2 the observed agreement rose to 0.89 and κ to 0.61 (95% CI 0.32–0.91); by V4 the observed agreement reached 0.96. Nucleocapsid IgG results had an observed agreement of 0.95 and a κof 0.64 (95% CI 0.18–1.00) at V1 (Table 2). At V2 the observed agreement was 0.94 and κ was 0.69 (95% CI 0.36–1.00), and by V4 the observed agreement was 0.82 and κ was 0.64 (95% CI 0.43–0.84).

**Table 2:** Saliva and DBS serologic test concordance for detection of SARS-CoV-2 infection among study participants, by specimen collection timepoint, US Naval Academy, Annapolis, Maryland, USA, August 2020–May 2021*

Serologic test	Aug 2020, n = 41			Dec 2020, n = 47			May 2021, n = 55	
	DBS-negative	DBS-positive		DBS-negative	DBS-positive		DBS-negative	DBS-positive
Spike IgG†								
Saliva-negative	31 (75.6)	5 (12.2)		37 (78.7)	5 (10.6)		0	2 (3.6)
Saliva-positive	1 (2.4)	4 (9.8)		0	5 (10.6)		0	53 (96.4)
Kappa coefficient (95% CI)	0.49 (0.15–0.83)			0.61 (0.32–0.91)			N/A	
Observed agreement	0.85			0.89			0.96	
Nucleocapsid IgG‡								
Saliva-negative	37 (90.24)	1 (2.4)		40 (85.1)	2 (4.3)		23 (41.8)	5 (9.1)
Saliva-positive	1 (2.4)	2 (4.9)		1 (2.1)	4 (8.5)		5 (9.1)	22 (40.0)
Kappa coefficient (95% CI)	0.64 (0.18–1.00)			0.69 (0.36–1.00)			0.64 (0.43–0.84)	
Observed agreement	0.95			0.94			0.82	

*Sample restricted to participants with both DBS and saliva specimens available. Values are no. (%) except as indicated. DBS, dried blood spot. †Receptor-binding domain target for salivary assay. ‡Salivary nucleocapsid IgG positivity defined as receptor-binding domain IgG and nucleocapsid IgG positivity.

**Figure 1:**
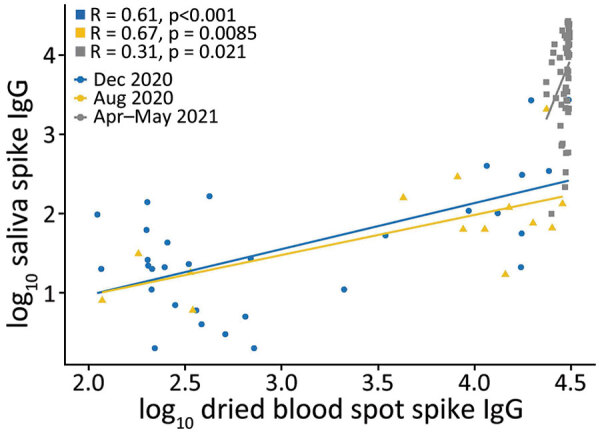
Quantitative comparison of spike IgG in saliva and dried blood spots among 79 study participants, US Naval Academy, Annapolis, Maryland, USA, December 2020–May 2021.

**Figure 2:**
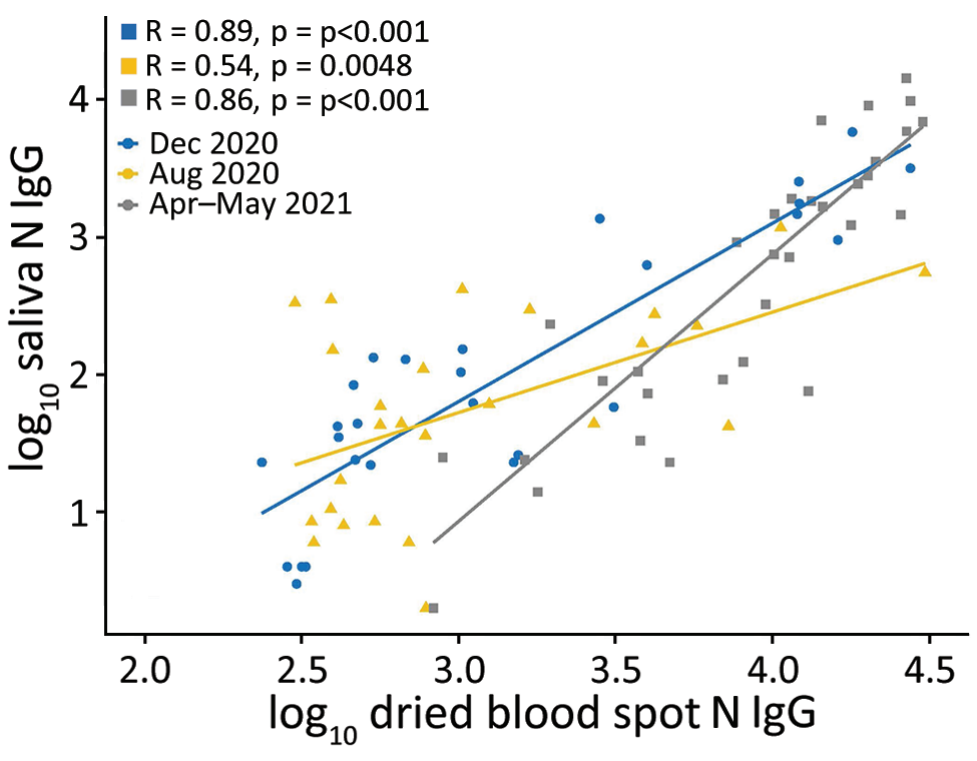
Quantitative comparison of nucleocapsid IgG in saliva and dried blood spots among 79 study participants, US Naval Academy, Annapolis, Maryland, USA, December 2020–May 2021. N, nucleocapsid.

Spike IgG MFI in saliva and DBS were significantly correlated at all 3 timepoints (Figure 1); high spike IgG values at V4 were consistent with the participants receiving vaccinations in March–April 2021. Nucleocapsid IgG MFI in saliva and DBS also were significantly correlated at all 3 timepoints (Figure 2).

## Conclusions

This study, conducted among a population of midshipmen at USNA in the first year of the COVID-19 pandemic, employed blood and saliva collection at multiple visits to evaluate the validity of salivary antibody surveillance. We observed concordance between DBS and saliva for the detection of spike and nucleocapsid IgG, and both biospecimen types were similar to RT-PCR for detection of cases. We noted that all vaccinees mounted a spike IgG response in DBS by V4, consistent with the known immunogenicity of these vaccines, but only 49.1% vaccinees had detectable nucleocapsid IgG at V4, indicating a substantive SARS-CoV-2 infection attack rate in the first half of 2021.

This assessment of SARS-CoV-2 detection in a congregate setting can help inform approaches for detection of SARS-CoV-2 in populations before and after vaccination. Prior evidence shows that PCR testing is an efficient method of infection control in congregate communities if administered regularly but that asymptomatic cases may still be undetected (*12*). These findings may apply to surveillance for other respiratory infections, such as influenza. A limitation to this study was the inability to directly match RT-PCR testing with blood and saliva collection, small sample size with paired samples, and loss to follow-up after the end of the academic year.

In summary, this assessment supports using saliva testing as a less invasive, more feasible surveillance method for monitoring changes in disease prevalence and susceptibility in large populations. Future directions include validation of alternative antibody targets, in both serum and saliva, which can discriminate antibody prevalence in the context of preexisting vaccination and postinfection hybrid immunity.

Appendix Additional information about evaluating SARS-CoV-2 saliva and dried blood spot surveillance strategies in a congregate population.
