# Supplementary material for: Evaluating SARS-CoV-2 Saliva and Dried Blood Spot Surveillance Strategies in a Congregate Population
Source: Emerg Infect Dis. 2023 Sep;29(9):1925–8. doi: 10.3201/eid2909.230417 (PMC10461675; doi:10.3201/eid2909.230417)
Supplement: Appendix — Additional information about evaluating SARS-CoV-2 saliva and dried blood spot surveillance strategies in a congregate population. [file 23-0417-Techapp-s1.pdf]

Article DOI: https://doi.org/10.3201/eid2909.230417

EID cannot ensure accessibility for supplementary materials supplied by authors. Readers who have difficulty accessing supplementary content should contact the authors for assistance.

Evaluation of SARS-CoV-2 Saliva and Dried Blood Spot Surveillance Strategies in a Congregate Population

Appendix

Additional Methods

Midshipmen were physically present at the United States Naval Academy from early August until mid-December (Fall Semester) and early January until late May (Spring Semester).

During the time of the study, non-pharmaceutical and other control interventions included: PCR testing of all Midshipmen at the beginning of each semester followed by 10 days of in-room isolation; randomly assigned weekly PCR testing of 15% of the asymptomatic Midshipman population, weekly PCR testing of all in-season varsity athletes; 10-day isolation for COVID-19 cases; close contact tracing, quarantine, and isolation; mask wear in all indoor environments aside from one’s dormitory room; increased cleaning of common surfaces and spaces; encouragement of frequent handwashing; social distancing of 6 feet; socially distanced eating; transition of all meetings to the online environment; separation from the community with decreased liberty leave to Annapolis; and transition of academic classes to a fully online or hybrid (50% in-person, 50% online) model.

Two of the 104 study participants were unvaccinated. One participant did not complete follow-up and the second seroconverted by the third scheduled visit, before the campus-wide vaccination campaign, based on saliva sampling.

Appendix Table. Timeline of data and sample collection for The Observational Seroepidemiologic Study of COVID-19 at the United States Naval Academy (TOSCANA)

Assessment	Enrollment (August-October)	Mid-Year Follow-up (December)	Winter break Follow-up (February)	End-of-Year Follow-up (April/May)
Informed Consent	X			
Demographics	X			
ARI* risk factors, ARI/COVID history, symptoms	X	X	X	X
Self-collected blood sample	X	X		X
Self-collected saliva sample	X	X	X	X

*Acute respiratory infection
